# A critical influence of HIF-1 on MMP-9 and Galectin-3 in oral lichen planus

**DOI:** 10.1186/s12903-024-04457-6

**Published:** 2024-06-29

**Authors:** Hala H. Hazzaa, Marwa A. M. El Shiekh, Osama Elkashty, Eman Magdy, Dalia Riad, Eman khalifa, Gasser M. Elewa, Naglaa M. Kamal

**Affiliations:** 1 Department of Oral Medicine, Periodontology and Diagnosis, Faculty of Dental Medicine for Girls, Al Azhar University, Cairo, Egypt; 2 Oral and Dental Biology Department, Faculty of Dental Medicine for Girls, Al Azhar University, Cairo, Egypt; 3 Department of Oral Pathology, Faculty of Dentistry, Mansoura University, Mansoura, Egypt; 4 Department of Oral Medicine, Diagnosis and Periodontology, Faculty of Dentistry, Beni_Suef University, Maadi, Cairo, Egypt; 5 Department of Oral Biology, Faculty of Dentistry, Beni_Suef University, Maadi, Cairo, Egypt; 6 Department of Cell Biology, Faculty of Oral and Dental Medicine, Delta University for Science & Technology, Dakhliya, Egypt; 7 Department of Oral Medicine, Periodontology, Diagnosis and Oral Radiology, Delta University for Science and Technology, Dakahlia, Egypt; 8 Department of Oral Pathology, Faculty of Oral and Dental Medicine, Ahram Canadian University, 6th of October, Cairo, Egypt

**Keywords:** Oral lichen planus, Hypoxia-inducible factor 1a, Galactin-3, MMP-9

## Abstract

**Objective:**

Oral lichen planus carries a risk for malignancy. The pathogenesis of the disease is mediated by various inflammatory mediators. Several mediators could be responsible for the oncogenic behavior in certain cases. Hypoxia-inducible factor-1a (HIF-1), and its possible correlation to Galactin-3 (Gal-3) and matrix metalloproteinase-9 (MMP-9) over expression represents an important indicator for malignant transformation. The investigation of these factors may present evidence-based information on malignant transformation of the disease.

**Subjects and methods:**

The study investigated the expression of HIF-1, Gla-3 and MMP-9 in tissue samples of OLP compared to control subjects of un-inflamed gingival overgrowth. 20 biospecimen were allocated in each group.

**Results:**

Immunohistochemical findings of OLP showed immunoreactivity for Galectin 3, HIF1a and MMP-9 by most of the epithelial cells. There was a positive correlation between HIF1α and MMP-9, *r* = 0.9301 (P-value < 0.00001). A positive correlation was detected between Galectin 3 and MMP-9, *r* = 0.7292 (P-value = 0.000264) between Galectin 3 and HIF1α, *r* = 0.5893 (P-value = 0.006252).

**Conclusion:**

These findings confirm the hypothesis that the adaptive pathways to hypoxia as Gal 3 and MMP-9 expressions and their HIF-1 may play a crucial role in carcinogenesis of OLP.

## Introduction

Oral lichen planus (OLP) is a chronic inflammatory immune-mediated disease that affects both, skin and oral mucosa. The disease is characterized by a T-cell-mediated immune response against epithelial cells, resulting in basal cell degeneration, basement membrane disruption, and T lymphocyte infiltration [1]. OLP is distinguished histo-pathologically by the presence of a band-like lymphocytic infiltrate at the interface between the epithelium and connective tissue, as well as the destruction of the basal layer [2]. Although its etiology and pathogenesis are not fully understood; some suggested etiologies include genetic predisposition, psychological alterations, neurological changes, and immunological changes [3]. Recent research has linked chronic inflammatory illnesses like OLP to hypoxia, which is the lack of adequate oxygen flow to a particular tissue [4]. However, research on how hypoxia may play a role in the pathogenesis of OLP is still insufficient.

Hypoxia-inducible factor 1a (HIF-1a) is activated under hypoxic conditions and subsequently regulates the expression of numerous transcription factors required for mediating adaptive responses to hypoxia [5]. HIF-1a has also been linked to the regulation of various cancer phenotypes such as cell proliferation, metastasis, and angiogenesis. The investigation of hypoxic and angiogenic-like proteins indicates that they may play a role in the pathogenesis of OLP. HIF-1 is a basic helix-loop-helix transcription factor composed of two subunits: HIF-1alpha and HIF-1beta [6]. HIF-1a protein overexpression has been linked to the pathogenesis of a variety of inflammatory and autoimmune diseases, including lupus nephritis [7] and rheumatoid arthritis [8]. In OLP, the over expression of HIF is associated with carcinomatous changes [6, 9].

Galectins have been implicated in cell modulation, immune system, inflammation, wound healing, and the progression of pathological conditions such as carcinogenesis [10]. Activated T cells, fibroblasts, epithelial cells, and tumor cells are just few examples of the diverse cell types that contain Gal-3. Gal-3 is a special member of the galectin family that is expressed by a single gene on chromosome 14. This mediator has been linked to a wide variety of biological processes, including the control of cell cycle and apoptosis as well as the progression and spread of cancer [11, 12]. Gal-3 overexpression was observed in hypoxic fields of cancer tissues, in this regard; the relationship between HIF-1a and Gal-3 in oral carcinogenesis was recently investigated [13, 14]. Researchers have proven a dynamic regulation of Gal-3 in response to the tumor microenvironment-associated hypoxia. In OLP, the over expression of HIF is associated with carcinomatous changes [9] and the over expression of matrix metalloproteinase-9 (MMP-9) was considered another proliferating-inducer in OLP as well [15].

Matrix Metalloproteinases (MMPs) are a group of at least 20 zinc-containing proteinases whose primary function is the proteolytic breakdown of proteins in connective tissue matrices. T cells from symptomatic OLP lesions contained higher levels of MMP-9 in culture precipitates when compared to those obtained from healthy controls’ peripheral blood [14]. Additionally, MMP-9 activators released by T cells activate pro MMP-9, resulting in the destruction of the epithelial basement membrane. The signal required for keratinocyte survival would not be provided by the damaged basement membrane in OLP lesions, leading to keratinocyte apoptosis. Moreover, MMP-9-induced basement membrane breakdown may allow antigen-specific CD8 + cytotoxic T cells to enter the OLP epithelium and cause further damage [15].

Curiously, MMP-9 was significantly expressed in response to the induced HIF-1a accumulation [16]. Given that HIF-1a induced miR-21 overexpression, preventing tumor cells from apoptosis in an oxygen-deficient environment in pancreatic cancer [17], a similar scenario of carcinogenesis is expected in symptomatic OLP categories. Furthermore, Gal-3, although present in the cytoplasm, nucleus and the cell surface, is also secreted into the extra-cellular matrix (ECM), where it binds to the ECM proteins. Cleavage of Gal-3 was reported to be initiated by the active form of MMPs; this step was reported as an active process during tumor progression; and Gal-3 could be hence used as a reliable marker for MMPs’ activities in growing breast cancers [18]. Based on the aforementioned conclusions, it can be presumed that MMP-9 may be responsible for a relative cleavage of Gal-3 in the ECM in symptomatic OLP lesions, which are carry the risk of malignancy; in a HIF-1a driven process. No experimental evidence was provided about this relation in symptomatic OLP so far. Therefore, in this new trial, the purpose was to analyze the expression of HIF-1a, MMP- 9 and Gal-3 in in biopsies diagnosed with symptomatic OLP.

## Materials and methods

### Biospecimen retrieval

The Oral lichen planus tissue specimens and the control oral mucosal specimen (gingival overgrowth) were retrieved from the biospecimen repository of the oral pathology department, Faculty of Oral and Dental Medicine, Al-Azhar University (Girls Branch), Cairo, Egypt.

### Ethical approval

This study was conducted during May 2023. The study protocol and consents were reviewed and approved by the Medical Ethical Committees, Al Ahram- Canadian University. Approval number: IRB00012891#45.

### Study design

This a cross-sectional analytical study- Immunohistochemical.

### Histopathology and immunohistochemistry

#### a. Tissue processing and section preparation

Control and OLP biopsies were fixed in 10% formalin, and then, embedded in paraffin blocks. Four sections with 4 μm thickness were taken from each block. Tissue staining was done as follows: The first section was stained with hematoxylin and eosin (H&E) for lichen planus diagnosis confirmation. Second section stained with Galectin 3 monoclonal antibody. Third section stained with HIF1a polyclonal antibody. Fourth section stained with MMP-9 polyclonal antibody.

Histo-pathologic examination of the slides was carried by blinded pathologist. Each specimen was examined by two examiners to provide a double-checked result. Any inter-examiner disagreement was solved by exposing the specimen to third examiner. All oral pathologists were set blind to the clinical diagnosis. All cases with doubtful diagnosis were excluded after histopathologic evaluation.

#### b. Immunohistochemical (IHC) staining

For IHC staining with Galectin 3, HIF1a, and MMP-9 antibodies, xylene deparaffinization of the sections was done, followed by rehydration in graded ethanol. Heat-induced retrieval of antigen was done by citrate buffer PH (6.0), then followed by blockage of the endogenous peroxidase activity by immersing the sections in hydrogen peroxide (H2O2). Washing in phosphate-buffered saline (PBS), and addition of reagent for protein blocking and incubation within humid chamber at 37 °C for 20 min for reducing the non-specific staining.

In the current study, the primary antibodies used were: Recombinant monoclonal rabbit antibody for Galectin 3 (Catalog No. A 22768 at dilution 1:100, ABclonal Technology USA). Polyclonal rabbit antibody for HIF1a (Catalog No. A7684 at dilution 1:100, ABclonal Technology USA). Polyclonal rabbit antibody for MMP-9 (Catalog No. A0289 at dilution 1:100, ABclonal Technology USA).

Overnight sections incubation with the primary antibody was done, followed by double washing in PBS and treatment with the labeled streptavidin-biotin complex (LSAB + System-HRP, Dako) at room temperature, for primary antibodies binding. Peroxidase activity visualization occurred by immersing the tissue sections in diaminobenzidine (Liquid DAB + Substrate, Dako), where a brown reaction product is shown. Finally, sections’ counterstaining was done using Mayer’s hematoxylin and then cover-slipped.

#### c. Immunohistochemical analysis

The immune-expression of Galectin 3, HIF1a, and MMP-9 was evaluated where the presence of brown colored immunostaining reaction in the nucleus or cytoplasm by using quantitative analysis. Five microscopic fields from each slide that show the highest immunopositivity were selected by two observers. Immunoreactivity was assessed in each microscopic field by estimating the area percentage of positive immune-stained cells in relation to the total area examined using computerized image analyzer composed of MIC-W16 digital camera installed on MEIJI MX5200L microscope, using a 200 X objective. The resulting 200 X images were analyzed on Intel® core I7® based computer using Fiji ImageJ (version 1.51r; NIH, Maryland, USA) software [19]. The image analyzer was calibrated automatically to convert the measurement units (pixels) produced by the image analyzer program into actual micrometer units. The area and area percentage reaction were measured. For each specimen, the mean values of the area percentage of positively stained cells with standard deviations were then determined.

### Sample size calculation

Sample size calculation was based on mean MMP-9 immunohistochemical expression in epithelium among studied groups retrieved according to Venktesh Naikmasur et al. [20] Using G*power version 3.1.9.4. to calculate sample size based on effect size of 0.6967, 2-tailed test, α error = 0.05 and power = 80.0% then total sample size will be 35 in each group.

### Statistical analysis

Data were analyzed using GraphPad Prism 8 (GraphPad Software). Quantitative data were described using mean, standard deviation for normally distributed data after testing normality using Shapiro-Wilk test. Significance of the obtained results was judged at the (0.05) level. Student unpaired t-test was used to assess the difference significance between the gingival overgrowth and lichen planus groups. Correlation between different parameters was done by using Pearson correlation test. To measure the strength of a linear association between two variables Pearson correlation coefficient was used where the value *r* = − 1 means a perfect negative correlation and the value *r* = 1 means a perfect positive one.

## Results

### Hematoxylin and eosin stain findings

**Fig. 1:**
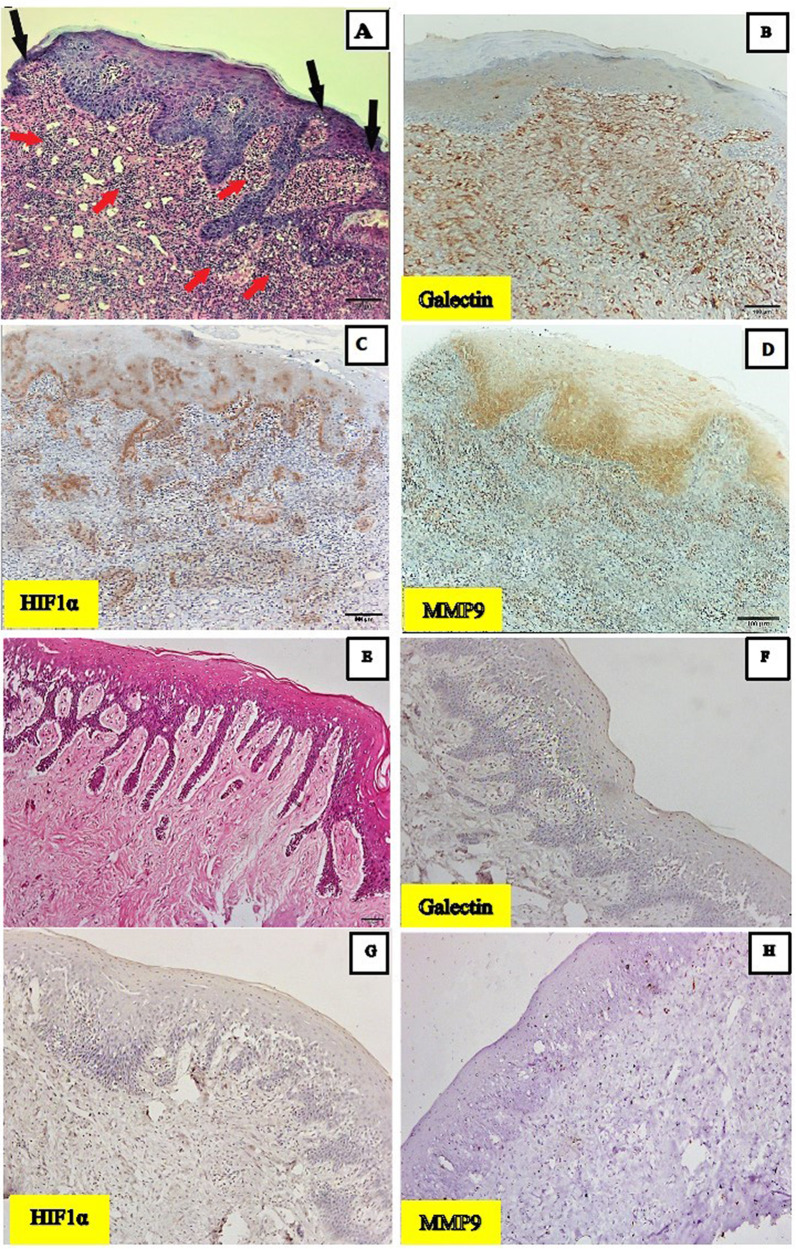
(**A**): A photomicrograph of Atrophic/erosive LP revealed areas with thinned epithelium (black arrows) and subepithelial dense lymphocytic infiltrate (red arrows). (H&E x100). (**B**): A photomicrograph of Galectin 3 immune reactivity in Atrophic/erosive LP showed positive reaction mainly in the subepithelial infiltrate of lymphocytes and in the epithelial cells of the rete ridges (Galectin 3 × 100). (**C**): A photomicrograph of HIF-1a immune reactivity in Atrophic/erosive LP showed positive reaction in the epithelial keratinocytes and the subepithelial infiltrate of lymphocytes (HIF-1a x100). (**D**): A photomicrograph of MMP-9 immune reactivity in Atrophic/erosive LP showed positive reaction by most of the epithelial cells mainly the basal layer and the subepithelial lymphocytic infiltrate (MMP-9 × 100). (**E**): A photomicrograph of gingival overgrowth revealed thickened keratinized stratified squamous epithelium with elongated rete ridges and normal lamina propria (H&E x100). (**F**): A photomicrograph of Galectin 3 immune reactivity in gingival overgrowth samples that showed a negative reaction (Galectin 3 × 100). (**G**): A photomicrograph of HIF-1a immune reactivity in gingival overgrowth samples that showed a negative reaction (HIF-1a x100). (**H**): A photomicrograph of MMP-9 immune reactivity in in gingival overgrowth samples that showed a negative reaction (MMP-9 × 100)

Atrophic/erosive OLP revealed areas with thinatrophic epithelium and subepithelial dense lymphocytic infiltrate (Fig. 1A). Samples of gingival overgrowth (control) revealed thick keratinized stratified squamous epithelium with elongated rete ridges with normal lamina propria (Fig. 1E).

### Immunohistochemical findings

All the samples of OLP showed immunoreactivity for Galectin 3 mainly in the sub-epithelial infiltrate of lymphocytes and in the epithelial cell layers. While negative expression was noted in gingival overgrowth samples (Fig. 1B, F).

Regarding HIF-1a, all OLP specimens showed mainly nuclear and some faint cytoplasmic immunopositivity in the epithelial keratinocytes and the sub-epithelial infiltrate of lymphocytes, while negative expression was noted in gingival overgrowth samples (Fig. 1C, G).

In oral lichen planus samples, immunopositivity for MMP-9 was identified by most of the epithelial cells mainly the basal layer cells and the sub-epithelial lymphocytic infiltrate. Samples of gingival overgrowth were negative for MMP-9 (Fig. 1D, H).

### Histo-morphometric findings

Regarding Galectin 3, the greatest mean area percentage of positively stained cells was recorded in the LP group compared to the control. The difference was statistically significant (*p* < 0.0001). The same for HIF-1a and MMP-9 as the greatest mean area percentage of positively stained cells was recorded in the LP group compared to the control. The difference was statistically significant (*p* < 0.0001) (Table 1).

**Table 1:** The comparison between the two studied groups regarding Galectin 3, HIF1a, and MMP-9 immuno-expression (histo-morphometric analysis)

	Galectin 3 area percent		HIF-1a area percent		MMP-9 area percent	
	Oral lichen planus	Gingival overgrowth	Oral lichen planus	Gingival overgrowth	Oral lichen planus	Gingival overgrowth
**Maximum**	15.84	4.367	22.69	2.224	42.96	1.224
**Minimum**	9.031	2.876	10.16	1.15	27.32	0.142
**mean ± SD**	11.8 ± 2.04	3.56 ± 0.75	15.76 ± 3.96	1.58 ± 0.57	34.04 ± 5.45	0.75 ± 0.55
**P-value**	< 0.0001*		< 0.0001*		< 0.0001*	

Significance level *p* < 0.05, *significant

SD, standard deviation

### The correlation between the three markers

Table 2 has shown the correlation findings between the three markers in OLP. The findings of this study have shown that there was a significant strong positive correlation between HIF-1a and MMP-9, *r* = 0.9301 (P-value < 0.00001). However, a significant moderate positive correlation was detected between Galectin 3 and MMP-9, *r* = 0.7292 (P-value = 0.000264) and between Galectin 3 and HIF-1a, *r* = 0.5893 (P-value = 0.006252).

**Table 2:** Correlation between the 3 markers in oral lichen planus

	MMP-9	Galectin 3
**HIF1a**	*R* = 0.9301 *P* < 0.00001*	*R* = 0.5893 *P* = 0.006252*
**Galectin 3**	*R* = 0.7292 *P* = 0.000264*	

Significance level *p* < 0.05, *significant

## Discussion

OLP meets all the requirements for hypoxia at the base of the angiogenic process triggered by proliferating inflammatory components [21].

There is growing evidence indicating that HIF-1a exerts significant and independent impacts on pathological angiogenesis. An increase in angiogenic factor has been linked to the hypoxic effect on the inflamed stromal area generated by an increase in the proliferating lymphocyte cellular mass in OLP samples [22].

Our results showed significant immune expression of HIF-1α in A-ELP compared to the non-inflamed gingival overgrowth. HIF-1a immune reactivity in erosive LP showed a positive reaction in the epithelial keratinocytes and the subepithelial infiltrate of lymphocytes. These were also reported by de Carvalho Fraga et al. [4] who indicated that HIF-1a is activated in the hypoxic condition in mammalian cells, and it then controls genes transcription in angiogenesis, erythropoiesis, glycolysis, iron metabolism, and cell survival. In addition, HIF-1a polymorphisms increase the expression of its target genes, so modifying the microenvironment and promoting the sequential release of inflammatory mediators in OLP. Hence, HIF-1a contributes significantly to the chronicity of oral mucosa lesions in OLP patients.

Moreover, according to the findings of Wang et al. [23] the expression of HIF-1a in OLP specimens was substantially higher than in normal oral mucosa tissues. They found that under hypoxic conditions, keratinocytes isolated from OLP tissues displayed a reduction of keratinocyte growth and elevated expression of HIF-1a, both of which may be involved in pathological alterations leading to the malignant transformation of OLP.

The diagnostic value of Gal-3 has been extensively investigated in various types of malignancies including thyroid carcinoma [24]. The significance of Gal-3 in inflammation and inflammatory disorders has been extensively reviewed [25]. The functional role of Gal-3 expression in various types of human cells denotes that it could be a possible diagnostic target [26]. Yet, little information is available in OLP.

Our study found a significant immune expression of Gal-3 in A-ELP compared to the gingival overgrowth. This is consistent with the findings of Ghapanchi et al. [27], who discovered substantial variations in serum Gal-3 (a member of the galectin family) levels between patients and healthy controls and proved Gal-3’s diagnostic capability for OLP. Also, erosive/atrophic forms had a higher Gal-3 concentration than reticular forms. Moreover, in accordance with our results, elevated expression of Gal-3 was demonstrated in different types of oral lesions. In patients with salivary gland tumors, high serum level of Gal-3 was detected, and could moreover differentiate patients from the healthy individuals [27].

MMP­9 is a gelatinase that plays a crucial role in both healthy and diseased inflammatory processes, where it aids in tissue remodeling. It is found in cytoplasmic granules of neutrophils and is a byproduct of macrophages. Moreover, stromal cells release it in response to inflammatory cytokines [28]. The production and release of MMP-9 by activated lymphocytes and monocytes are tightly controlled by inflammatory cytokines [28].

Discontinuity of basement membrane possibly leads to apoptosis of keratinocytes, which is typical in OLP, and this is connected to the activity of MMPs. Previous studies stated that there might be a link between MMPs and OLP. In a study by Zahou et al. in 2001, squamous cell carcinoma and OLP have higher MMPs expression compared to normal [17]. OLP has lower MMPs expression than carcinoma, but it is still higher than normal. These findings indicate two aspects, MMPs can not only have an impact on disease pathogenesis, but they can also encourage more invasive behavior later in the disease’s course, resulting in the transformation of OLP to SCC. Zhou et al. [15], reported that higher expression of MMP-9 in inflammatory infiltrates was possibly due to enhanced release of the enzyme from T‑cells inside the infiltrate.

The results revealed a significant increase in immune expression of MMP-9 by epithelial cells mainly basal layer and the sub-epithelial infiltrate of lymphocytes in A-ELP compared to non-inflamed gingival overgrowth. This was in accordance with the results of Paulusová et al. [29] who showed that MMP-9 expression is most prominent in the region of lymphocytic inflammatory infiltration in the lamina propria, which includes lymphocytes within the overlaying epithelium, in all cases of OLP. Furthermore, the findings of Zhou et al. [15] who found that MMP-9 was higher in OLP than in healthy control patients, support this theory.

Here in, a positive correlation was noticed between Gal-3 and MMP-9 (at *r* = 0.7292). Given that cleavage of Gal-3 can be initiated by the active form of MMPs [15]; MMP-9 may be therefore an active member responsible for this process in symptomatic OLP patients, a novel finding for further future studies. A positive correlation was also evident in our results between HIF-1a and MMP-9 (at *r* = 0.9301). This can be explained in the light of the conclusive findings of Wan et al. [18] who reported that he induction of MMP-9 immuno-expression occurs secondary to HIF-1a accumulation. Taken together, an organized HIF-1a driven process is now highly expected to modulate first the MMP-9 expression, which will subsequently feed the Gal-3 cleavage and expression in A-ELP categories with their expected insult in the malignant transformation sequelae of such subtypes; in agreement with our novel hypothesis.

### Limitations

Authors believe that the present study sample size may limit the generalization of the study results. However, the findings suggested a potential role of the investigated biomarkers and further clinical trials are suggested.

## Conclusion

Within the limits of this clinical report, the revealed observations shed a light on the importance of the histopathological tissue changes, specifically hypoxia and chronic inflammation, in controlling the carcinogenic behavior of OLP. This confirms the hypothesis that the adaptive pathways to hypoxia as Gal-3 and MMP-9 expressions and their HIF-1a mediated up-regulation pathway may play a crucial role in carcinogenesis. Consequently, it would be of great interest to see whether inhibition of HIF-1a can give rise to beneficial therapeutic efficiency in symptomatic OLP lesions with dysplastic changes, for future prospective studies.

## Data Availability

All data are available by the main author, Dr. Hala Hazzaa upon request.
